# Symptoms and diagnostic criteria of acquired Megacolon - a systematic literature review

**DOI:** 10.1186/s12876-018-0753-7

**Published:** 2018-01-31

**Authors:** Tahleesa Cuda, Ronny Gunnarsson, Alan de Costa

**Affiliations:** 1 Cairns Clinical School, College of Medicine and Dentistry, James Cook University, 451 Draper Street, Cairns, QLD 4870 Australia; 2 Department of Surgery, Cairns Private Hospital, Cairns, QLD Australia; 3 Research and Development Unit, Primary Health Care and Dental Care, Southern Älvsborg County, Cairns, Region Västra Götaland Sweden; 4 Department of Public Health and Community Medicine, Institute of Medicine, The Sahlgrenska Academy, University of Gothenburg, Cairns, Sweden

**Keywords:** Acquired, Idiopathic, Megacolon, Redundant, Symptoms

## Abstract

**Background:**

Acquired Megacolon (AMC) is a condition involving persistent dilatation and lengthening of the colon in the absence of organic disease. Diagnosis depends on subjective radiological, endoscopic or surgical findings in the context of a suggestive clinical presentation. This review sets out to investigate diagnostic criteria of AMC.

**Methods:**

The literature was searched using the databases - PubMed, Medline via OvidSP, ClinicalKey, Informit and the Cochrane Library. Primary studies, published in English, with more than three patients were critically appraised based on study design, methodology and sample size. Exclusion criteria were studies with the following features: post-operative; megarectum-predominant; paediatric; organic megacolon; non-human; and failure to exclude organic causes.

**Results:**

A review of 23 articles found constipation, abdominal pain, distension and gas distress were predominant symptoms. All ages and both sexes were affected, however, symptoms varied with age. Changes in anorectal manometry, histology and colonic transit are consistently reported. Studies involved varying patient numbers, demographics and data acquisition methods.

**Conclusions:**

Outcome data investigating the diagnosis of AMC must be interpreted in light of the limitations of the low-level evidence studies published to date. Proposed diagnostic criteria include: (1) the exclusion of organic disease; (2) a radiological sigmoid diameter of ~ 10 cm; (3) and constipation, distension, abdominal pain and/or gas distress. A proportion of patients with AMC may be currently misdiagnosed as having functional gastrointestinal disorders. Our conclusions are inevitably tentative, but will hopefully stimulate further research on this enigmatic condition.

## Background

For a condition that is sometimes treated surgically, Acquired Megacolon (AMC) is poorly understood and diagnostic criteria remain obscure [1–6]. The term refers to a colon of increased diameter and increased length, in the absence of organic disease [7–9]. The colon is often described as distended or dilated [10–13]. Some authors differentiate ‘colonic redundancy’ from ‘megacolon’, by describing increased colonic length with reduplication, opposed to a colon of increased diameter [9, 14–16]. However, increased length and diameter often co-exist. The definition AMC used in this paper encompasses both increased colonic length and diameter with negligible rectal involvement where possible.

Many symptoms including constipation, distension, abdominal pain and a poor sense of wellbeing are attributed to AMC [17]. Pathophysiology, natural history and effective symptom management, although speculated, are unknown [3, 7, 16, 18–24]. A large body of evidence exists, including literature reviews, which have analysed surgical outcomes for AMC and the diagnosis of idiopathic megabowel (megarectum-inclusive disease) [1, 3, 4]. However, consensus on diagnostic criteria is still lacking.

The objective of this systematic literature review is to refine diagnostic criteria for this condition and to evaluate symptoms and pathophysiology that may be associated with AMC.

## Methods

The review protocol is available on the University of York Centre for Reviews and Dissemination database PROSPERO; registration number CRD42014013307; registration date 28/08/2014. The processing and reporting of this review are consistent with the general recommendations provided by the PRISMA revision [25].

The following online databases were searched electronically: PubMed, Medline via OvidSP, ClinicalKey, Informit and the Cochrane library. The search terms used were “acquired megacolon”, “idiopathic megacolon”, “dolichocolon” and “redundant colon”. Two independent reviewers developed inclusion and exclusion criteria.

The selection criteria were primary studies:

1. Diagnosing AMC using radiological, histological, laparoscopic or open surgical, endoscopic or other means;
2. Investigating the symptomatology and presentation of AMC; and
3. Providing pre-operative data.

The exclusion criteria were:

1. Primary studies with exclusively post-operative data;
2. Acquired megarectum-predominant disease;
3. Exclusively paediatric studies;
4. Studies of organic or obstructive causes of megacolon;
5. Animal models;
6. Studies that failed to exclude organic causes of megacolon;
7. Studies with less than three patients; and
8. Full text not available.

Studies published in English, from randomised controlled trials, non-randomised trials, cohort studies or case series consisting of three or more patients were selected. As there were few studies meeting these criteria no limit was put on date of publication.

Kantor (1924) failed to definitively exclude organic causes for megacolon. Despite satisfying a component of exclusion criteria, this primary publication was referenced by nearly every other study included in this review. Deemed a vital contributor to the study of AMC, both reviewers allowed the inclusion of this study in the review [7].

Factors including study design, year of publication, numbers of patients, controls and methods used to exclude organic disease were recorded. Themes relating to diagnostic criteria, colonic dimensions, histology, colonic transit time and anorectal manometry were recorded, as were patient demographics.

Mean, range, standard deviation and statistical significance were pooled and provided in the review. Conclusive findings were discussed where available. Meta-analysis were not performed due to the heterogeneity of the studies. Authors agreed to exclude individual patients with incomplete data and rectal predominant disease.

## Results

**Fig. 1:**
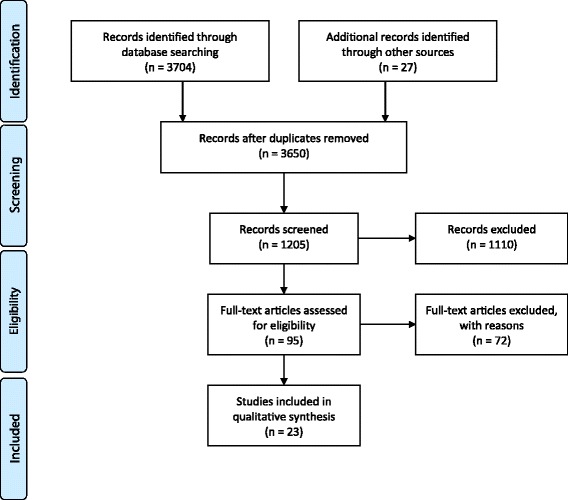
PRISMA 2009 flow diagram

The literature search identified 1205 publications of potential interest and 23 of these fulfilling inclusion and exclusion criteria (Fig. 1), described 532 patients with AMC (Table 1). A slight female preponderance was observed with a mean age of 52 years (Table 2).

**Table 1:** Characteristics of Studies Investigating the Diagnosis of AMC

Authors	Year	Study Design	No. pts	Control
Kantor [7]	1924	Cohort	62	606
Kobak et al. [30]	1962	Case reports	3	0
Rios-Dalenz et al. [31]	1975	Case series	49	25
Goulston [40]	1976	Cohort	82	52
Lane and Todd [19]	1977	Case series	42	0
Taylor et al. [26]	1980	Cohort	15	12
Ryan [29]	1982	Cohort	6	60
Preston et al. [9]	1985	Cohort	20	50
Barnes et al. [20]	1986	Case series	10	0
Stabile et al. [3]	1991	Case series	40	0
Koch et al. [35]	1993	Cohort	6	13
Koch et al. [34]	1996	Cohort	10	10
Gattuso et al. [36]	1996	Case series	3	10
Gattuso et al. [22]	1997	Cohort	48	0
Gattuso et al. [32]	1997	Cohort	6	17
Gattuso et al. [27]	1998	Cohort	1	44
Chen et al. [33]	2002	Cohort	5	24
Lee et al. [23]	2005	Cohort	9	10
Meier-Ruge et al. [37]	2006	Case series	63	21
Wedel et al. [38]	2006	Cohort	8	12
Iantorno et al. [24]	2007	Cohort	9	10
Yoshino et al. [28]	2007	Cohort	4	0
Ohkubo et al. [39]	2014	Cohort	31	16
Total			532	992

*Pts* Patients, *No.* number

**Table 2:** Age and Sex Distribution of Patients Studied with AMC

Authors	Pub. date	No. pts	Age mean	Age range	Female	Male
Kantor [7]	1924	62			24	38
Kobak et al. [30]	1962	3	61	48–68	0	3
Lane and Todd [19]	1977	42			21	21
Preston et al. [9]	1985	20			9	11
Koch et al. [35]	1993	6	55	31–76	4	2
Koch et al. [34]	1996	10	64	31–98	4	6
Gattuso et al. [22]	1997	48		12–69	22	26
Gattuso et al. [32]	1997	6		34–66	3	3
Gattuso et al. [27]	1998	1	37	37	0	1
Chen et al. [33]	2002	5		24–41	1	4
Lee et al. [23]	2005	9	30	10–66	2	7
Meier-Ruge et al. [37]	2006	63		15–75	54	9
Wedel et al. [38]	2006	8		2–58		
Iantorno et al. [24]	2007	9		39–68	3	6
Yoshino et al. [28]	2007	4	57	29–74	1	3
Ohkubo et al. [39]	2014	31	52	19–83	22	9
Total		327	52	2–98	170	149

*Pts* Patients, *mo* Months, *y* age in years

Weighted mean

### Exclusion of organic disease

All studies, with the exception of Kantor (1924), excluded organic disease by demonstrating an intact anorectal inhibitory reflex, the absence of hypoganglionosis on rectal biopsy or a combination of the two. Two studies showed an abnormal anorectal inhibitory reflex but normal histology [19, 20]. The anorectal inhibitory reflex was present in all patients in eight studies [3, 9, 19, 22–24, 26, 27]. Three of four patients of Yoshino et al. (2007) described intact anorectal inhibitory reflexes [28]. Twenty-one of 26 patients of Gattuso et al. (1997) had an intact anorectal inhibitory reflex [22]. One study in an endemic area for trypanosoma Cruzii performed three consecutive pathology screens to exclude Chagas Disease [24].

### Physical dimensions

A variety of studies recorded colonic diameters of patients with AMC or provided dimensions used to diagnose its presence (Table 3). Recent contributions on this topic emphasise colonic diameter as a defining feature [9, 19, 26, 27, 29–32]. Earlier studies referred to length [7, 30, 31, 33]. Twelve studies used barium enema or similar studies [3, 7, 9, 19, 27, 29, 30, 32, 34, 35]. Taylor et al. (1980) was the only study to use abdominal X-rays [26].

**Table 3:** Imaging Findings Used to Diagnose AMC

Author	Pub. Date	No. Pts	Physical Characteristics Observed in Patients Diagnosed with AMC	Modality
Lane and Todd [19]	1977	42	Partial or total dilatation large bowel	BE
Ryan^a^ [29]	1982	6	Patients dilated colon proximal to sigmoid	BE/AXR
Preston et al. [9]	1985	20	Rectal diameter level S2 10+/− 3 cm (mean) Rectal diameter level pelvis brim 9.5+/− 2.4 cm (mean) Sigmoid diameter 10+/− 3.5 cm (mean) Descending diameter 7.2+/− 2.1 cm (mean) Transverse diameter 7.8+/− 1.4 cm (mean) Ascending diameter 8.2+/− 1.6 cm (mean) Conclusion that sigmoid width at pelvic brim greater than 6.5 cm abnormal	DCBE
Stabile et al. [3]	1991	40	Subjective proximal colonic enlargement and loss of haustral pattern Descending colon “usually” greater than 6 cm Ascending colon “usually” greater than 8 cm	BE
Koch et al. [35]	1993	6	Sigmoid diameter 6.5-15 cm (range), 10 cm (mean)	AXR
Gattuso et al. [36]	1996	3	Rectal diameter 4-11 cm (range) Colon diameter 6-10 cm (range)	
Meier-Ruge et al. [37]	2006	63	Distal and proximal colon circumference hemicolectomy 9-12 cm	Specimen
Iantorno et al. [24]	2007	9	Sigmoid diameter 10+/− 2 cm (mean)	BE

*Pts* Patients, *DCBE* Double-contrast barium enema, *BE* Barium enema, *AXR* Abdominal X-Ray

^a^ amongst patients with volvulus

Using 50 double contrast barium enema studies, Preston et al. (1985) concluded a normal diameter of the rectosigmoid at the pelvic brim was less than 6.5 cm [9]. Six studies of this review used this criterion to diagnose AMC [23, 27, 32–35]. The three studies, Preston et al. (1985), Iantorno et al. (2007) and Koch et al. (1993) calculated mean sigmoid diameters of 10+/− 3.5 cm, 10+/− 2 cm and 10 cm respectively for sigmoid-inclusive AMC [9, 24, 35].

The presence of colonic reduplications, angulations or loops seen during barium studies or enema-filled sigmoid loops rising above the iliac crests was described by Kantor (1924) [7]. Taylor et al. (1980) diagnosed AMC by an increased colonic diameter on radiological imaging and chronic constipation [26]. Ryan (1982) described the recurrence of both colonic dilatation and symptoms in patients who had undergone segmental colectomy for megacolon volvulus of previously non-dilated segments [29].

Kantor (1924) demonstrated the limitations of simple X-ray techniques, with individual colonic dimensions varying significantly with serial imaging amongst individuals [7].

### Symptoms

Ten studies investigated symptoms associated with AMC, for which eight are tabulated [3, 7, 19, 21–24, 27] (Table 4). Most predominant symptoms were constipation, abdominal pain, distention and gas distress.

**Table 4:** Occurrence of Symptoms in Patients with a Diagnosis of AMC

Authors	Year	No. pts	Const.	Diarr.	Faecal incont.	Vomit.	Gas distress	Abdo. pain	Dist.	Faecal impact.	Painful evac.	Digital evac.	Abdo. mass	Pseudo-obstruct.
Kantor [7]	1924	62	48	16		14	45	36						
Lane and Todd [19]	1977	42	42		13			21	12					
Stabile et al.^a^ [3]	1991	40	40		14			34	40	14	16	8	14	
Basilisco et al. [21]	1996	14	8						12					4
Gattuso et al. [22]	1997	48				1		35	24	13		1		
Gattuso et al. [27]	1998	1	1					1	1					
Lee et al. [23]	2005	9	6					1						
Iantorno et al. [24]	2007	9	9											
Total (mean %)		225	154 (68%)	16	27	15	45	128 (57%)	89 (40%)	27	16	9	14	4

*No* number, *pts.* patients, *const* constipation, *diarr* diarrhea, *incont* incontinence, *vomit* vomiting, *abdo* abdominal, *dist* distension, *impact* impaction, *evac* evacuation, *obstruct* obstruction

^a^ includes patients with whole colon and left colon only involvement

Both Lane and Todd (1977) and Stabile et al. (1991) reported adults presenting with constipation, distension and abdominal pain while children presented with faecal impaction and soiling [3, 19]. The study by Barnes et al. (1986) described both patients with AMC and acquired-megarectum (AMR) with distal colonic involvement. AMC-specific data could not be clearly extrapolated from those with AMR. Therefore, it could not be included in the final data tabulation. This study reported symptoms in children with early onset (i.e. less than 10 years old) as faecal soiling, constipation, distension, abdominal pain, rectal impaction and abdominal mass on palpation. Patients with onset of symptoms later in life (i.e. greater than 10 years old) had constipation, distension and abdominal pain [20]. Kantor (1924) estimated that 23% of patients with constipation have AMC [7].

### Colonic histology

Histological findings were presented in 11 papers [23, 24, 27, 30, 32, 34–39] (Table 5). As different techniques, sample sizes and sites were used, no definite conclusions could be drawn. A general theme of enteric architectural and neurochemical abnormality in patients with AMC was inferred.

**Table 5:** Histopathological Findings of AMC

Author	Date	No. Pts	Findings
Kobak et al. [30]	1962	2	1× nerve ganglia in rectal segment distal to megacolon, some diminution of ganglia in involved area 1× nerve ganglia observed entire colon
Koch et al. [35]	1993	6	1× CM hypertrophy 2× LM hypertrophy Diminished concentrations AChE in ME *p* < 0.01 Diminished concentrations VIP in ME *p* = 0.03 Diminished VIP-staining neurons CM + LM Diminished immunostaining neuronal bodies SPE Myenteric plexus, submuocus plexus externus, submucous plexus internus normal No inflammatory infiltrate
Koch et al. [34]	1996	10	Diminished concentration VIP ME p = 0.01 Increased NADPH diaphorase activity in ME *p* = 0.01
Gattuso et al. [36]	1996	3	Hypertrophy ME Lower density NADPH-diaphorase in ME Decreased neural density shown by PGP 9.5 immunoreactivity Smaller number nitric oxide motor system nerve fibres in ME
Gattuso et al. [32]	1997	6	No thickening enteric smooth muscle No change in density of enteric innervation 2× mild melanosis coli 3× mild chronic inflammatory cell infiltrate LP 2× hypertrophied ME No thickening enteric smooth muscle *p* < 0.005 2× fibrosis LM 1× fibrosis CM 3× fibrosis MM
Gattuso et al. [27]	1998	1	Hypertrophy LM Hypertrophy MM Diminished enteric neural density in ME, most marked in LM Increased density AChE− + ve nerves in LP Mild-moderate fibrosis ME
Lee et al. [23]	2005	9	Diminished ICC and PGP 9.5 reactive neuronal structures in all colonic layers *p* < 0.05
Meier-Ruge et al. [37]	2006	63	Atrophied collagenous tendinous connective tissue membrane of MP Atrophied tendinous fibre net of MP Type III collagen absent from MP ICC/collagen II/collagen IV/smooth muscle actin/desmin/fibronectin no consistent alteration
Wedel et al. [38]	2006	8	SMMHC, HDAC8 and/or SM absent of lacking in 75% Diminished myofilaments of myocyte clusters Oligoneuronal hypogangliosis
Iantorno et al. [24]	2007	9	Decreased enteric neurons and enteric glial cells Decreased ICC but increased ICC-IM Diminished NSE − +ve and S100 − +ve cells in SP and MP 67% lymphocyte infiltration MP and SP *p* = 0.13
Ohkubo et al. [39]	2014	31	Non-dilated loops exhibited similar histopathologic findings as dilated loops 61.3% damaged and/or severe reduction ganglion cells 35.5% atrophy and/or vacuolar degeneration of smooth muscle cells of MP 32.2% abnormal ICC network 19.4% atrophy and/or vacuolar degeneration of smooth muscle cells of MP and damaged and/or severe reduction ganglion cells 3.2% atrophy and/or vacuolar degeneration of smooth muscle cells of MP and abnormal ICC network 12.9% damaged and/or severe reduction ganglion cells and abnormal ICC network

*Pts* Patients, *CM* Circular muscularis externa, *LM* Longitudinal muscularis externa, *AChE* Acetyl cholinesterase, *ME* Muscularis externa, *VIP* Vasoactive intestinal peptide, *NADPH* Nicotinamide adenine dinucleotide phospate diaphorase, *LP* Lamina propria, *MM* Muscularis mucosa, *ICC* Interstitial cells of Cajal, *PGP* protein gene product neuronal marker, *MP* Myenteric plexus, *SMMHC* smooth muscle myosin heavy chain smooth muscle marker, *HDAC8* histone deacetylase 8 smooth muscle marker, *SM* smoothelin smooth muscle marker, *SP* Submucosal plexus, *NSE* Neuron specific elonase enteric neuronal marker, S100, Schwann cell marker IM, intramuscular space

Three studies demonstrated decreased concentration of interstitial cells of Cajal, [23, 24, 39] three suggesting diminished ganglia, [30, 38, 39] four with diminished enteric neural densities [23, 27, 35, 38] and three suggesting enteric smooth muscle hypertrophy [27, 32, 35]. Conversely, other studies showed normal enteric neuron histology and enteric muscle thickness [3, 19, 20].

Koch et al. (1993) and Koch et al. (1996) investigated colonic wall neurotransmitters and enzyme systems in AMC. Conclusions made from these studies include decreased vasoactive intestinal peptide (VIP) and acetylcholinestrase (AChE) activity in the muscularis externa and increased nicotinamide adenine dinucleotide phosphate (NADPH) diaphorase activity in the muscularis externa [34, 35]. Koch et al. (1993) suggested a hypothesis for the development of AMC involving: (1) colonic hypertrophy resulting from prolonged cholingeric nerve mediated contractions of the circular muscularis externa; or (2) colonic dilatation secondary to prolonged contraction of the longitudinal smooth muscle [35].

Okhubo et al. (2014) demonstrated that non-dilated colonic loops exhibited similar histopathological abnormalities as dilated loops in AMC and that histopathological abnormalities preceded clinical symptoms in some circumstances [39]. Genomic sequencing by Chen et al. (2002) found no mutation of neurturin [33].

### Anorectal manometry

Techniques used to study anorectal pressures varied. One study found resting anal canal pressures in AMC were higher than controls [26]. Half of patients of Taylor et al. (1980) recorded anal canal pressures higher among AMC than controls [26]. Yoshino et al. (2007) found patients with AMC had a higher incidence of very slow anal pressure waves [28].

Rectal sensation was decreased in 50% and normal in 50% of patients with AMC in the study by Lane and Todd study (1977). Increased rectal capacity was also noted [19]. Chen et al. (2002) described rectal hyposensation in four of five AMC cases [33]. Diminished rectal sensitivity to balloon distension but intact perianal sensation and rectal electrosensation was described by Koch et al. (1997) [22].

### Colonic transit time

Prolonged colonic transit times were recorded in majority of patients by Chen et al. (2002) and Stabile et al. (1991) [3, 33]. The study by Yoshino et al. (2007) demonstrated very slow periodical pressure changes in the anal canal signifying ultra-slow colonic transit time (OR 10.67, 95% CI 4.40–25.86) [28].

### Complications

Five studies reported incidence of volvulus, colonic obstruction or faecal impaction with AMC [22, 30, 31, 39, 40]. Sigmoid volvulus occurred as follows: Kobak et al. (1962) two patients; Rios-Dalenz et al. (1975) 49 patients; Ohkubo et al. (2014) two patients and Gattuso et al. (1997) five patients [22, 30, 31, 39]. Goulston (1976) described a 6.6:1 rate of impacted faeces, megacolon and volvulus among a psychiatric hospital patients in comparison to a general hospital [40].

### Neuropsychiatric conditions

Six studies showed an association of AMC with neurological disease [3, 28, 30, 34, 35, 40]. Goulston et al. (1976) reported faecal impaction, volvulus and megacolon were higher in a psychiatric hospital, in comparison to a general hospital, 0.53% to 0.08% respectively [40].

Three studies recorded psychiatric conditions, predominantly schizophrenia, among their patients [30, 34, 35]. Cerebrovascular accidents and other organic central neurological conditions such as epilepsy were also of note [3, 28, 30, 34, 35]. Two patients of Gattuso et al. (1997) suffered mental retardation [22].

## Discussion

This review sought to evaluate the diagnosis of AMC. Twenty-three studies were identified, 17 of which had control patients. The sample size in many of these studies was small and few studies provided the statistical significance of their findings. Conclusions drawn are inevitably tentative.

### Demographics

Patients with AMC may present at in both genders and at any age, though the presentation of children differ. There may be an association with neuro-psychiatric conditions and medications used in this patient group. It is unknown if the condition is a result of inappropriate behavioural response to defaecation, enteric physiological impairment or is associated with the use of medication.

### Symptoms

The common features of an adult presentation were constipation, distension, gas distress and abdominal pain. In comparison, children presented with faecal incontinence and impaction.

No study assessed the impact on quality of life. There may well be a considerable overlap between the symptoms associated with AMC and Constipation Predominant Irritable Bowel Syndrome given 20% of constipated patients have an AMC [7]. Brummer et al. (1962) supports this, estimating that 30% of patients with constipation have an AMC [16]. Whether patients with AMC are being misdiagnosed as having Constipation Predominant IBS cannot be deduced from this review.

### Imaging

Imaging, usually barium enema, has been used. The bowel appears as a grossly elongated and dilated colon with multiple loops [3, 9, 19, 24, 29, 35–37]. The Preston et al. (1985) study, referenced by six studies in this review, proposes that rectosigmoid diameter at the pelvic brim exceeding 6.5 cm as diagnostic [9, 23, 27, 32–35]. Gladman et al. (2007) reported weaknesses with this study involving their choice of control group, despite its use by many later reports [1]. A mean sigmoid diameter around 10 cm is suggested. [9, 24, 35] The sigmoid is often involved radiologically, [9, 19, 24, 29, 30, 35] but AMC may exclusively affect the proximal colon, [3, 9, 29, 41] for which this definition is not suitable.

Simple 2D imaging has limitations in assessing colonic diameters and variations occur between serial images [7]. There seems little doubt that the increasing availability of CT colonography using standardised insufflation pressure will optimise the diagnosis of AMC [42]. CT colonography has the advantage of being a quantitative imaging modality and offers an alternative approach to evaluate the colon and rectum following incomplete colonoscopy [43, 44]. Low radiation dose imaging is also possible with modern scanners [45]. CT Colonography allows simple measurement of colonic diameters and length from multiple views, has shorter procedure times, does not require recovery supervision and carries less procedural risks than traditional colonoscopy [42, 44, 46–49]. It currently has a prominent role following incomplete colonoscopy - a common occurrence amongst patients with AMC. The use of this modality is limited in screening as biopsy and polypectomy cannot be performed [44].

### Colonoscopy

Many colonoscopists seem confident about diagnosing AMC or colonic redundancy, although no objective criteria for diagnosis have been defined [46, 50, 51]. This modality, does however, depend upon subjective interpretation. AMC or colonic redundancy have been associated with incomplete colonoscopy, as a result, may not be the most appropriate investigation for this population group [46, 52]. Hanson et al. (2007), analysed the colonic length of patients with redundant colon during colonoscopy using CT colonography. This study reported patients with incomplete colonoscopy as having colonic lengths exceeding 200 cm, often reported as redundant colon during colonoscopy [46]. Colonic redundancy was defined as elongated and tortuous colons or those with two or more acute flexures [46, 53]. Although the diagnosis of AMC was not pursued in this study, it may suggest that CT colonography is a useful modality to diagnose abnormal colonic dimensions, both in terms of length, diameter and possibly volume [46].

### Pathophysiology

While no definitive consensus of histological or neurochemical changes was achieved, the study by Koch et al. (1993) suggests plausible mechanisms for the development of AMC and warrant further investigation [35]. Consecutive studies in this review conclude with findings of altered neurochemical and enteric architectural findings [34, 35].

AMC is a disease of exclusion. Ruling out an organic cause for this condition is pertinent. The absence of hypogangliosis has more consistent results when compared with anorectal reflex testing, although it does carry more risk.

### Complications

The incidence of colonic volvulus in AMC may be underestimated in this review, as colectomy studies were largely excluded. Emergency colectomy is indicated for patients presenting acutely with volvulus as a consequence of AMC. In the elective setting, surgeons may encounter patients with AMC experiencing intractable symptoms and poor quality of life that have failed conservative management. For the surgeon, the decision to operate must be balanced against the risk of not optimising their symptoms. Amongst this demographic, elective colectomy is sometimes offered as a final attempt.

## Conclusion

Based on the data collated in this review, we propose the following criteria for the diagnosis of acquired megacolon: (1) the exclusion of organic disease by rectal biopsy or an intact anorectal inhibitory reflex; (2) a sigmoid diameter of ~ 10 cm on abdominal X-ray or barium enema; (3) and symptoms including constipation, distension, abdominal pain and gas distress. Given the limitations of 2D radiological imaging, CT colonography may be a more optimal imaging modality.

The condition affects both sexes and has preponderance for the middle age [3, 7, 9, 19, 20, 22–24, 26–40]. Histologically, evidence supports enteric architectural and neurochemical abnormalities, however, detailed findings are variable [23, 24, 27, 30, 32, 34–39]. A proportion of patients with AMC may experience colonic volvulus [22, 30, 31, 39, 40]. Furthermore, the condition may have an increased prevalence among patients with neuropsychiatric conditions [3, 28, 30, 34, 35, 40].

Whether AMC is a single entity or a group of heterogeneous conditions is unknown. Neither is its relationship to other constipation predominant conditions. It may well be that patients with AMC are misdiagnosed as having Constipation Predominant IBS. The natural history of this condition and optimal forms of management are yet to be elucidated. Surgical procedures are performed on patients with AMC for intractable disease and emergency situations, risking morbidity and mortality [1, 4]. This systematic review may help in the understanding of the presentation, methods of diagnosis and some of the associations of AMC. Further research is required on the pathophysiology of the condition, protocols for conservative treatment and the place of surgery for intractable disease.

## Abbreviations

AMC: Acquired megacolon
AMR: Acquired megarectum
CI: Confidence interval
IBS: Irritable bowel disease
OR: Odds ratio

## References

[1] Gladman MA, Knowles CH (2008). Novel concepts in the diagnosis, pathophysiology and management of idiopathic megabowel. Colorectal disease : the official journal of the Association of Coloproctology of Great Britain and Ireland.

[2] Kamm MA, Stabile G (1991). Management of idiopathic megarectum and megacolon. Br J Surg.

[3] Stabile G, Kamm MA, Hawley PR, Lennard-Jones JE (1991). Colectomy for idiopathic megarectum and megacolon. Gut.

[4] Gladman MA, Scott SM, Lunniss PJ, Williams NS (2005). Systematic review of surgical options for idiopathic Megarectum and Megacolon. Ann Surg.

[5] Loder P (1998). Colorectal diseases: comment. Ausr NZ J Surg.

[6] Gattuso JM, Kamm MA (1993). Review article: the management of constipation in adults. Aliment Pharmacol Ther.

[7] Kantor J. A clinical study of some common anatomical abnormalities of the colon. American Roentgenray Society. 1924;

[8] Ewing M (1975). Dolichocolon. Aust, NZ J Surg.

[9] Preston DM, Lennard-Jones JE, Thomas BM (1985). Towards a radiologic definition of idiopathic megacolon. Gastrointest Radiol.

[10] Melling J, Makin CA (2011). Sigmoid volvulus, acquired megacolon and pseudo-obstruction. Surgery (Oxford).

[11] Pereira J, Horrigan F: Understanding adult acquired Megacolon. Geriatr Nurs 1987, January/February:16–19. 10.1016/s0197-4572(87)80183-0 3643149

[12] Adad S, Souza M, Silva G, do Carmo J, de Godoy C, Micheletti A (2008). Acquired non-Chagas megacolon associated with the use of psychiatric medication: case report and differential diagnosis with Chagas megacolon. Rev Soc Bras Med Trop.

[13] Bharucha AE, Phillips SF (1999). Megacolon: acute, toxic, and chronic. Current treatment options in gastroenterology.

[14] Liu R, Lin M, Yeh S (1989). Dolichocolon: an incidental finding on gallium Scintigraphy. Clin Nucl Med.

[15] Kantor J (1928). The common affections of the colon, their origin and their management. Bull N Y Acad Med.

[16] Brummer P, Seppala P, Wegelius U (1962). Redundant colon as a cause of constipation. Gut.

[17] Autschbach F, Gassler N (2007). Idiopathic megacolon. Eur J Gastroenterol Hepatol.

[18] Burrell ZL (1957). Acquired megacolon in the insane. Gastroenterology.

[19] Lane RH, Todd IP (1977). Idiopathic megacolon: a review of 42 cases. Br J Surg.

[20] Barnes PR, Lennard-Jones JE, Hawley PR, Todd IP (1986). Hirschsprung's disease and idiopathic megacolon in adults and adolescents. Gut.

[21] Basilisco G, Velio P, Bianchi PA (1997). Oesophageal manometry in the evaluation of megacolon with onset in adult life. Gut.

[22] Gattuso JM, Kamm MA (1997). Clinical features of idiopathic megarectum and idiopathic megacolon. Gut.

[23] Lee J, Park H, Kamm M, Talbot I (2005). Decreased density of interstitial cells of Cajal and neuronal cells in patients with slow-transit constipation and acquired megacolon. J Gastroenterol Hepatol.

[24] Iantorno G, Bassotti G, Kogan Z, Lumi CM, Cabanne AM, Fisogni S, Varrica LM, Bilder CR, Munoz JP, Liserre B (2007). The enteric nervous system in chagasic and idiopathic megacolon. Am J Surg Pathol.

[25] Moher DLA, Tetzlaff J, Altman DG, The PRISMA group (2009). Preferred reporting items for systematic reviews and meta-analyses: the PRISMA statement.

[26] Taylor I, Hammond P, Darby C (1980). An assessment of anorectal motility in the management of adult megacolon. Br J Surg.

[27] Gattuso JM, Smith VV, Kamm MA (1998). Altered contractile proteins and neural innervation in idiopathic megarectum and megacolon. Histopathology.

[28] Yoshino H, Kayaba H, Hebiguchi T, Morii M, Hebiguchi T, Ito W, Chihara J, Kato T (2007). Multiple clinical presentations of anal ultra slow waves and high anal pressure: megacolon, hemorrhoids and constipation. Tohoku J Exp Med.

[29] Ryan P (1982). Sigmoid volvulus with and without megacolon. Dis Colon rectum.

[30] Kobak MW, Jacobson MA, Sirca DM (1962). Acquired megacolon in psychiatric patients. Dis Colon rectum.

[31] Rios-Dalenz J, Smith L, Thompson T (1975). Diseases of the colon and Rectum in Bolivia. Am J Surg.

[32] Gattuso JM, Kamm MA, Talbot JC (1997). Pathology of idiopathic megarectum and megacolon. Gut.

[33] Chen B, Knowles CH, Scott M, Anand P, Williams NS, Milbrandt J, Tam PK (2002). Idiopathic slow transit constipation and megacolon are not associated with neurturin mutations. Neurogastroenterology and motility : the official journal of the European Gastrointestinal Motility Society.

[34] Koch TR, Schulte-Bockholt A, Otterson MF, Telford GL, Stryker SJ, Ballard T, Opara EC (1996). Decreased vasoactive intestinal peptide levels and glutathione depletion in acquired megacolon. Dig Dis Sci.

[35] Koch TR, Schulte-Bockholt A, Telford GL, Otterson MF, Murad TM, Stryker SJ (1993). Acquired megacolon is associated with alteration of vasoactive intestinal peptide levels and acetylcholinesterase activity. Regul Pept.

[36] Gattuso JM, Hoyle CH, Milner P, Kamm MA, Burnstock G (1996). Enteric innervation in idiopathic megarectum and megacolon. Int J Color Dis.

[37] Meier-Ruge WA, Muller-Lobeck H, Stoss F, Bruder E (2006). The pathogenesis of idiopathic megacolon. Eur J Gastroenterol Hepatol.

[38] Wedel T, Van Eys GJ, Waltregny D, Glenisson W, Castronovo V, Vanderwinden JM (2006). Novel smooth muscle markers reveal abnormalities of the intestinal musculature in severe colorectal motility disorders. Neurogastroenterology and motility : the official journal of the European Gastrointestinal Motility Society.

[39] Ohkubo H, Masaki T, Matsuhashi N, Kawahara H, Yokoyama T, Nakajima A, Ohkura Y (2014). Histopathologic findings in patients with idiopathic megacolon: a comparison between dilated and non-dilated loops. Neurogastroenterology and motility : the official journal of the European Gastrointestinal Motility Society.

[40] Goulston E (1976). Diverticular disease of the colon and megacolon. Incidence in a psychiatric centre compared with a teaching hospital. Med J Aust.

[41] Min BH, Son HJ, Kim JJ, Rhee JC, Lee SJ, Rhee PL (2010). Idiopathic proximal hemimegacolon: radiologic findings and analyses of clinical and physiological characteristics. Abdom Imaging.

[42] Patrick JL, Bakke JR, Bannas P, Kim DH, Lubner MG, Pickhardt PJ (2015). Objective volumetric comparison of room air versus carbon dioxide for colonic distention at screening CT colonography. Abdom Imaging.

[43] Horvat N, Raj A, Ward JM, Smith JJ, Markowitz AJ, Gollub MJ. Clinical value of CT Colonography versus preoperative colonoscopy in the surgical Management of Occlusive Colorectal Cancer. AJR Am J Roentgenol. 2017:1–8. 10.2214/AJR.17.18144 PMC7473453 29261351

[44] Weinberg DS, Pickhardt PJ, Bruining DH, Edwards K, Fletcher JG, Gollub MJ, Keenan EM, Kupfer SS, Li T, Lubner SJ, et al. Computed tomography Colonography vs colonoscopy for colorectal cancer surveillance after surgery. Gastroenterology. 2017; 10.1053/j.gastro.2017.11.025 PMC5847443 29174927

[45] Taguchi N, Oda S, Imuta M, Yamamura S, Nakaura T, Utsunomiya D, Kidoh M, Nagayama Y, Yuki H, Hirata K, et al. Model-based iterative reconstruction in low-radiation-dose computed tomography Colonography: preoperative assessment in patients with colorectal cancer. Acad Radiol. 2017; 10.1016/j.acra.2017.10.008 29191684

[46] Hanson ME, Pickhardt PJ, Kim DH, Pfau PR (2007). Anatomic factors predictive of incomplete colonoscopy based on findings at CT colonography. AJR Am J Roentgenol.

[47] Mang T, Graser A, Schima W, Maier A (2007). CT colonography: techniques, indications, findings. Eur J Radiol.

[48] Flor N, Rigamonti P, Pisani Ceretti A, Romagnoli S, Balestra F, Sardanelli F, Cornalba G, Pickhardt PJ (2013). Diverticular disease severity score based on CT colonography. Eur Radiol.

[49] Lips LM, Cremers PT, Pickhardt PJ, Cremers SE, Janssen-Heijnen ML, de Witte MT, Simons PC. Sigmoid Cancer versus chronic Diverticular disease: differentiating features at CT Colonography. Radiology. 2014:132829. 10.1148/radiol.14132829 25426771

[50] Schembre DB, Ross AS, Gluck MN, Brandabur JJ, McCormick SE, Lin OS (2011). Spiral overtube-assisted colonoscopy after incomplete colonoscopy in the redundant colon. Gastrointest Endosc.

[51] Tan EJ, Soh KC, Ngiam KY (2013). Colonic architectural change on colonoscopy in patients taking psychotropic medications. Surg Endosc.

[52] Yucel C, Lev-Toaff AS, Moussa N, Durrani H (2008). CT Colonography for incomplete or contraindicated optical colonoscopy in older patients. Am J Roentgenol.

[53] Galambos A, Galambos W (1946). Redundancy of the colon. American Journal of Digestive Disease.

